# Supplementary material for: Protein trafficking and synaptic demand configure complex and dynamic synaptome architectures of individual neurons
Source: Sci Rep. 2026 Mar 2;16:11541. doi: 10.1038/s41598-026-40513-7 (PMC13057022; doi:10.1038/s41598-026-40513-7)
Supplement: Supplementary file 1 — Supplementary Information. [file 41598_2026_40513_MOESM1_ESM.pdf]

Protein trafficking and synaptic demand configure complex and dynamic synaptome architectures of individual neurons

Supplementary Figure 1:

Model of a pyramidal CA1 neuron colour coded by values for normalised PSD95 puncta density taken on days 0 through day 7 of the Halo-ligand injection (from Bulovaite et al., 2022). See also main manuscript, figure 1A.

Supplementary Figure 2:

A. Distribution of the difference between a and b (a-b) rendered along the dendritic tree, shown along the normalised puncta density values at Day0 and Day7. It can be seen that a>b in most compartments and takes the max value in the most distal regions of the neuron (CA1lsms).

B. Same distribution as in A with the Demand values shown

Supplementary Figure 3:

Distribution of average value for difference between *a* and *b* trafficking rate constants *(a-b)* for each of 20 segments of the dendritic tree as described in manuscript.

Supplementary Table 1:

List of parameters used in three main versions of the model (each with increasing granularity): Version I – Three compartments with one degradation value and three local demand values; Version II – Three compartments with three degradation and three local demand values; Version III – 20 compartments with 20 local demand values and a linear model for degradation. Parameters are classified as global or local / subregion-specific (i.e. dependent on local demand). The column “fitting” indicates where parameter values were obtained by fitting the model to experimental data.

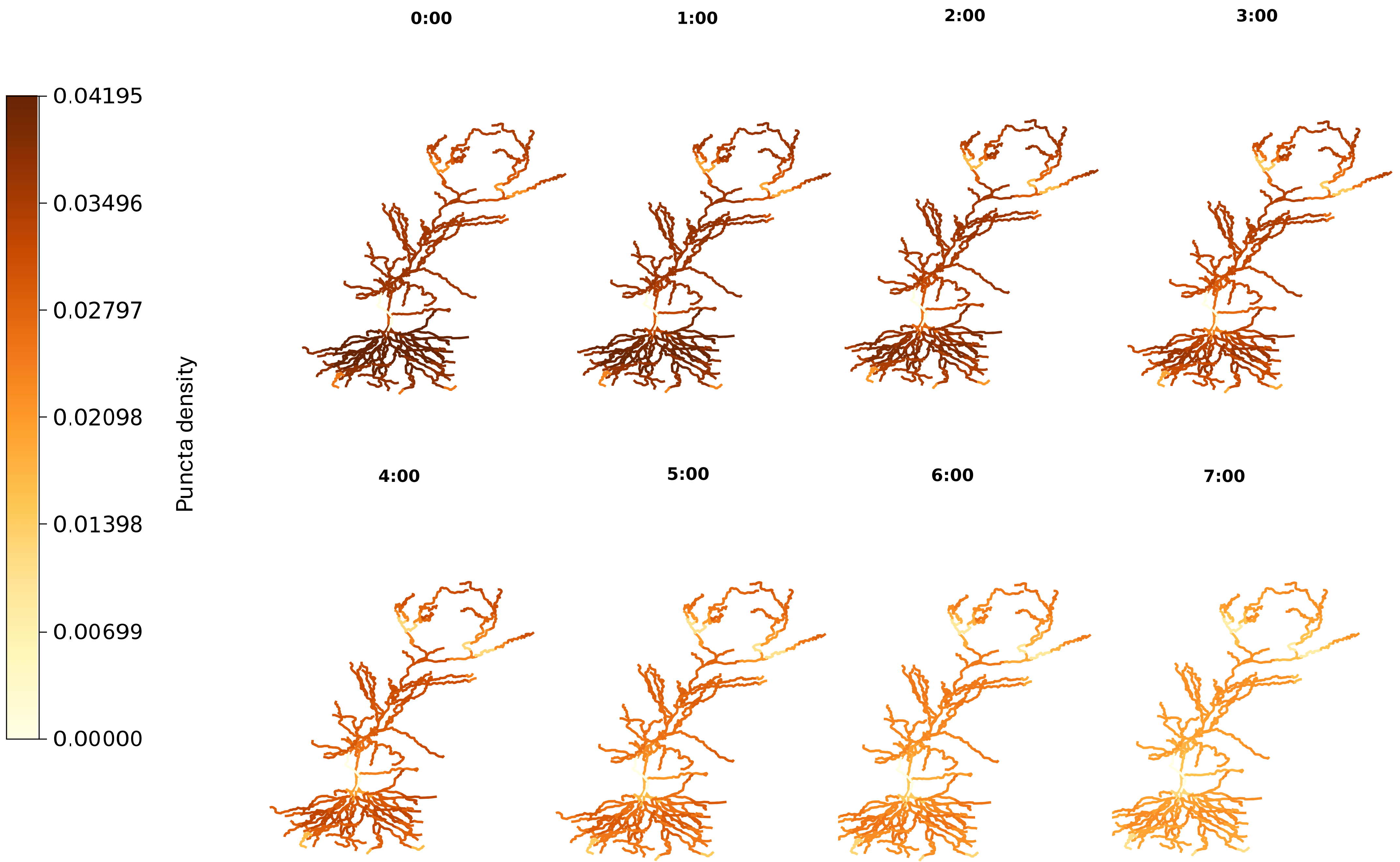
0:00
1:00
2:00
3:00
4:00
5:00
6:00
7:00
0.04195
0.03496
0.02797
0.02098
0.01398
0.00699
0.00000
Puncta density

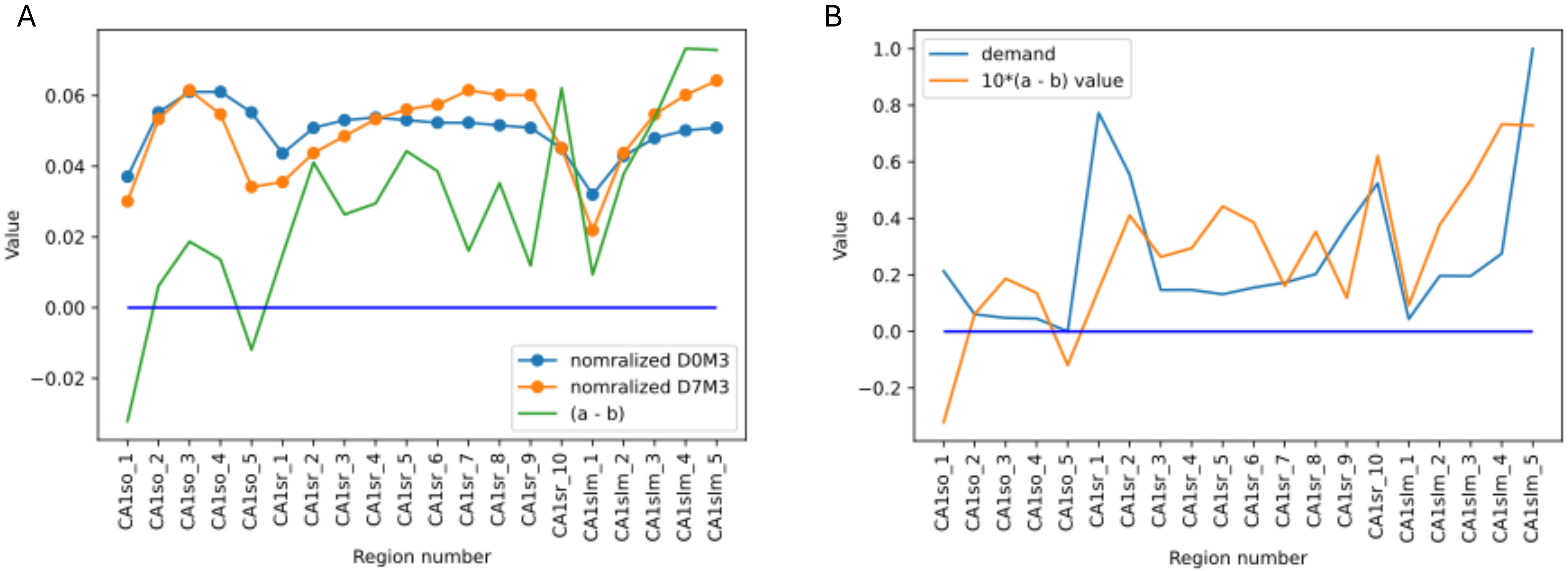
A
B
Value
Region number
nomralized D0M3
nomralized D7M3
(a - b)
demand
10*(a - b) value
0.06
0.04
0.02
0.00
−0.02
1.0
0.8
0.6
0.4
0.2
0.0
−0.2
CA1so_1
CA1so_2
CA1so_3
CA1so_4
CA1so_5
CA1sr_1
CA1sr_2
CA1sr_3
CA1sr_4
CA1sr_5
CA1sr_6
CA1sr_7
CA1sr_8
CA1sr_9
CA1sr_10
CA1slm_1
CA1slm_2
CA1slm_3
CA1slm_4
CA1slm_5

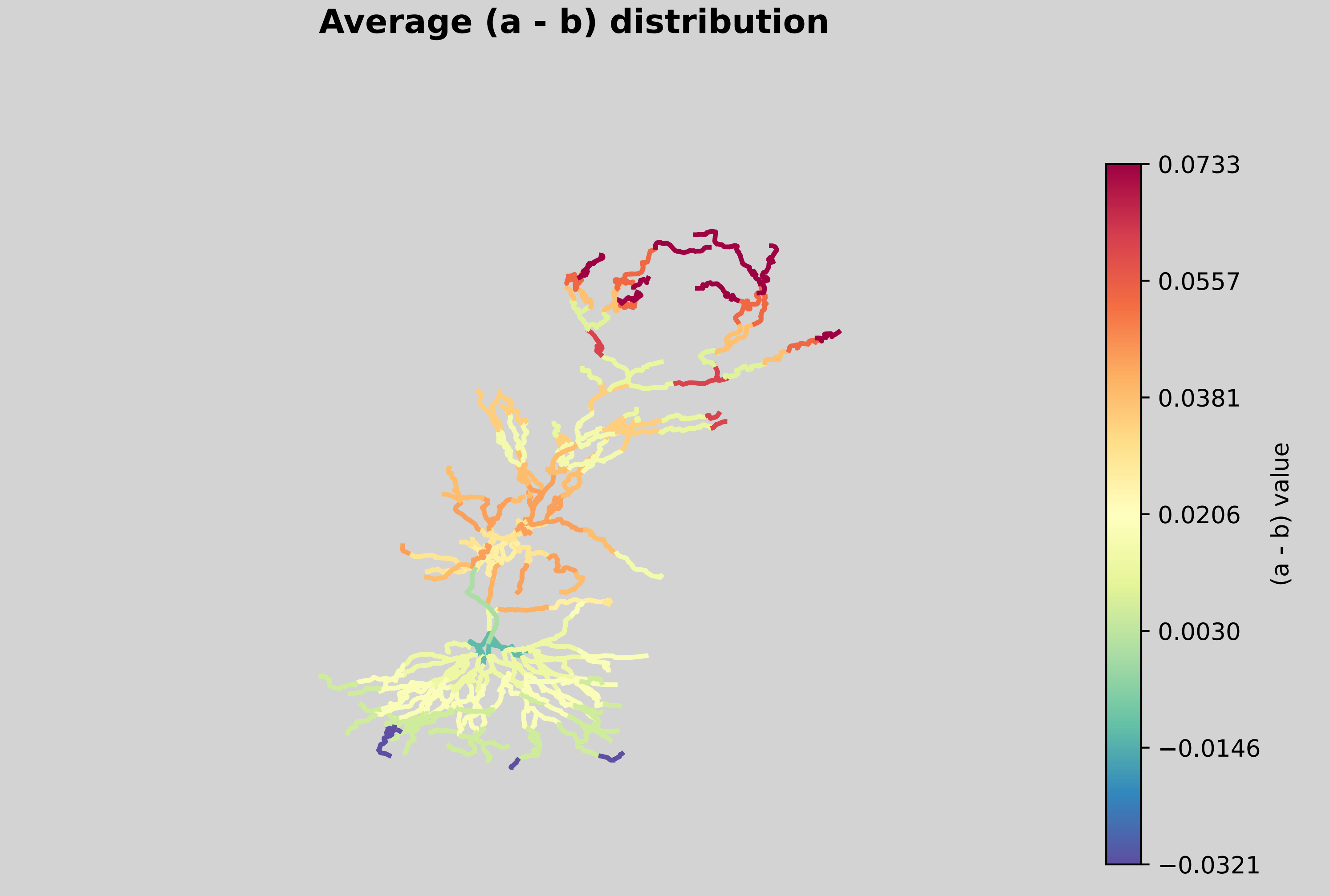

Average (a - b) distribution
0.0733
0.0557
0.0381
0.0206
0.0030
−0.0146
−0.0321
(a - b) value

Parameter name	Definition	Dimension	Model version			Global/Local	Fitting
			I	II	III		
u	concentration of PSD95 protein, attached to microtubules	dimensionless	YES	YES	YES	Local	NO
u*	concentration of the protein detached from microtubules	dimensionless	YES	YES	YES	Local	NO
**a**	rate of forward traffic	1/s	YES	YES	YES	Local	NO
**b**	rate of backword traffic	1/s	YES	YES	YES	Local	NO
**c**	rate of detachment	1/s	YES	YES	YES	Local	NO
**d**	rate of degradation	1/s	YES	YES	NO	Local	YES
**dvA**	Intercept for degradation linear approximation	1/s	NO	NO	YES	Global	YES
**dvB**	Slope for degradation linear approximation	1/s	NO	NO	YES	Global	YES
**F**	Ratio between DDT (1) and DDD (0) modes	dimensionless	YES	YES	YES	Global	YES
**Ctau**	Coefficient for demand-dependent detachment	dimensionless	YES	YES	YES	Global	YES
**mProp**	Ratio between attached and detached protein pools	dimensionless	YES	YES	YES	Global	YES
**Demand_CA1i** (**$\tilde{u}_i$ in [13]**)	Local demand value for each compartment: 3 values for Model 1 and II, and 20 for Model III	dimensionless	YES	YES	YES	Local	YES
